# Effects of high-intensity aerobic exercise on psychotic symptoms and neurocognition in outpatients with schizophrenia: study protocol for a randomized controlled trial

**DOI:** 10.1186/s13063-015-1094-2

**Published:** 2015-12-08

**Authors:** John A. Engh, Eivind Andersen, Tom L. Holmen, Egil W. Martinsen, Jon Mordal, Gunnar Morken, Jens Egeland

**Affiliations:** Division of Mental Health and Addiction, Vestfold Hospital Trust, Tønsberg, Norway; Faculty of Humanities and Education, Department of Practical, Physical and Aesthetic Education, Buskerud and Vestfold University College, Borre, Norway; Division of Mental Health and Addiction, Oslo University Hospital, Institute of Clinical Medicine, University of Oslo, Oslo, Norway; Department of Neuroscience, Faculty of Medicine, Norwegian University of Science and Technology (NTNU), Trondheim, Norway; Department of Psychiatry, St. Olav’s University Hospital, Trondheim, Norway; Department of Psychology, University of Oslo, Oslo, Norway

**Keywords:** High-intensity interval training, Schizophrenia, Psychosis, Neurocognition

## Abstract

**Background:**

The focus in recent years on physical inactivity and metabolic disturbances in individuals with schizophrenia raises the question of potential effects of physical activity. Physical activity has shown beneficial effects on cognition in healthy older individuals as well as on symptom severity in depression. However, opinions diverge regarding whether aerobic high-intensity interval training reduces cognition and key symptoms in schizophrenia. The main objective for the trial is to investigate the potential effects of aerobic high-intensity interval training on neurocognitive function and mental symptoms in outpatients with schizophrenia.

**Methods/Design:**

The trial is designed as a randomized controlled, observer-blinded clinical trial. Patients are randomized to 1 of 2 treatment arms with 12-week duration: aerobic high-intensity interval training or computer gaming skills training. All participants also receive treatment as usual. Primary outcome measure is neurocognitive function. Secondary outcome measures will be positive and negative symptoms, wellbeing, tobacco-smoking patterns and physiological/metabolic parameters. Patient recruitment takes place in catchment area-based outpatient clinics.

**Trial registration:**

ClinicalTrials.gov NCT02205684. Registered 29 July 2014.

## Background

Schizophrenia is a severe mental illness characterized by delusions and hallucinations (i.e. positive symptoms), affect flattening, poverty of speech, lack of motivation and social withdrawal (i.e. negative symptoms) and cognitive impairment, making it one of the leading causes of disability in the age group 15–44 years [1–3].

People with schizophrenia are more likely to smoke [4], to be physically inactive [5], suffer from malnutrition due to an unhealthy diet [6] and have low cardiorespiratory fitness (CRF) [7–9]. They commonly also suffer from comorbid psychiatric disorders such as depression as well as substance misuse [10] and manifest multiple somatic comorbidities [11]. Compared to healthy individuals there is a 2.5 to 4.0-fold increase in prevalence of the metabolic syndrome [12–14] and a 20 % reduction in life expectancy in individuals with schizophrenia.

Antipsychotic medication is a cornerstone in the treatment of schizophrenia, substantially reducing symptom severity and relapses [15]. However, antipsychotics are most effective in reducing positive symptoms, and they have minimal effect on negative symptoms or cognitive function [16]. Psychosocial interventions (e.g. cognitive behavior therapy, cognitive remediation therapy, family psychoeducation, social skills training, supported employment) are usually provided in addition to pharmacological treatment and play an important role in the treatment of schizophrenia, especially in improving daily life functioning.

Among healthy individuals, physical activity has positive effects on physical health, mental health and cognition. Moreover, positive effects of physical activity have also been described for persons with mental disorders, such as depression [17–20]. High-intensity interval training is shown to increase CRF and reduce the risk of various somatic illnesses [21, 22] and is established as a safe and useful exercise method. For people with schizophrenia evidence is sparse with regard to the effect of physical activity, in particular on post-exercise psychological states or neurocognition. A recent randomized controlled trial (RCT) investigated the effect of an aerobic exercise program in 33 individuals with schizophrenia (aerobic exercise, *n* = 16; treatment as usual, *n* = 17) [23]. After completion of the 12-week exercise program (1 hour each session, 3 times weekly) the aerobic exercise group showed a significant improvement compared to the control group. The effects of combined interventions were examined in a non-randomized study of 43 individuals with schizophrenia [24]. The participants were exposed to either endurance training (*n* = 22) or a control condition playing table soccer (*n* = 21). During the last half of the 12-weeks intervention the individuals in both groups also attended a cognitive remediation program. Significant improvements were found in short-term and long-term verbal memory and cognitive flexibility from the start of the combined intervention at week 6 to the end of the 3-month training period in the group exposed to endurance training augmented with cognitive remediation. However, conclusions concerning the effects of each component in the combined intervention are hard to draw due to the design of the study.

Recent reviews have called for well-designed studies to explore these important topics further [25–27]. Interpretation of previous studies investigating effects of regular sport activities lasting for weeks or months have been hampered by small sample sizes [28–30]. In the largest study to date, 20 patients with schizophrenia met compliance demands and received exercise therapy 1 to 2 hours a week for 6 months in the experimental group. When compared to 19 patients in the control condition receiving occupational therapy [8] significant effects were found on positive and depressive symptoms, as well as trend level decrease for negative symptoms in per protocol analyses. The total attrition rate was 38 %, and in the intention-to-treat analysis no significant effects were found.

The cardiovascular fitness hypothesis [31] suggests that CRF is a physiological mediator that explains the various mental health benefits of physical activity. In regard to cognitive functions, CRF might be more beneficial for individuals with limits on their cognitive reserves [32], such as people with schizophrenia. A finding of exercise-induced increase in hippocampal volume in patients with schizophrenia and a positive correlation of volume increase and short-term memory improvement supports this hypothesis [33].

Low CRF increases the risk of cardiovascular disease (CVD) [34, 35], which is the largest single cause of death in schizophrenia [36]. This, taken together with an unhealthy lifestyle, underscores the need for multi-disciplinary treatment and underlines the need for addressing physical activity and, in particular, high-intensity interval training, which is more effective than moderate intensity exercise in increasing CRF in the short term [37, 38].

Many exercise intervention studies for persons with mental disorders have methodological limitations, which makes it difficult to draw firm conclusions. The most common limitations are small sample sizes, that the randomization process has not been undertaken at a site remote from where the intervention takes place, lack of blinded assessment of outcome, imprecise assessments (if any) of CRF, low adherence to the exercise protocols, lack of long- term follow-up assessment, and not analyzing the data according to the intention-to-treat principle [39].

There is currently insufficient evidence to know whether regular high-intensity interval training reduces key symptoms in schizophrenia. Studies with larger sample sizes comprised of participants adhering to the exercise protocol are needed.

We aim to conduct a trial where these methodological considerations are addressed by comparing a 12-week aerobic high-intensity interval training (HIIT) program to skills training of the same duration, with regard to: 1) aspects of cognitive function, especially memory, attention and executive function, and 2) psychiatric symptom load (negative and positive symptoms) and wellbeing. Post-treatment improvement in cognition and psychotic symptoms are anticipated. We hypothesize that the individuals in the Exercise Group (EG) will perform better than individuals in the Computer Skills Group (CSG) on aspects of cognitive function, especially memory, attention and executive function. In addition, we expect ameliorated positive and negative symptoms in the EG.

The effects of HIIT on maximum oxygen uptake and risk factors for CVD (i.e. tobacco- smoking, elevated blood pressure, dyslipidemia, insulin resistance and high body fat) are also subject to investigation.

## Methods

### Trial design

**Fig. 1:**
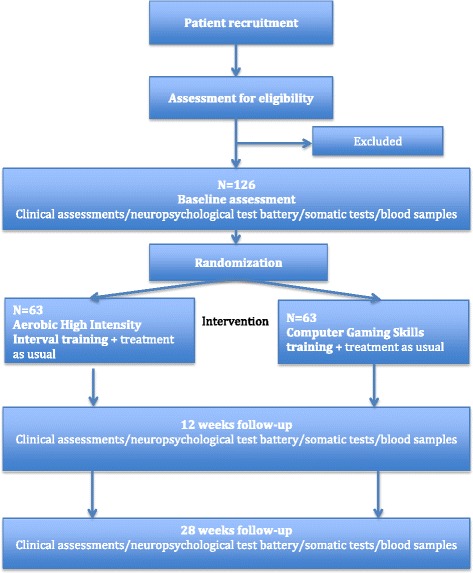
Flow chart – participants in the Effects of Physical Activity in Psychosis (EPHAPS) trial

This trial is designed as a randomized controlled, parallel group, observer-blinded clinical trial (Fig. 1).

Participants are randomized to either a high-intensity interval group (Exercise Group, EG) or to a comparison group (Computer Gaming Skills Group, CSG). During the 12-week interventions treatment as usual is continued for all patients. Patients are assessed at the start (T_0_) and end of the intervention (T_1_) as well as at 4 months post-treatment (T_2_). The trial is observer-blinded, as measurement of psychiatric symptoms and neurocognitive function is performed by research staff blinded for group allocation. The researchers are geographically dispersed from the training facilities. When assessed after training, the participants are instructed not to disclose what activity they have taken part in. The physiological testing is performed by staff involved in delivery of the interventions and will not be blinded except at baseline. The same holds for collecting self-ratings involving measures of activity.

### Recruitment and eligibility

The participants are outpatients at the psychiatric rehabilitation clinics in Tønsberg (Nordre Vestfold distriktspsykiatriske senter, NVDPS) and Larvik (Søndre Vestfold distriktspsykiatriske senter, SVDPS) in Vestfold County. The clinics are part of the Division of Mental Health and Addiction in Vestfold Hospital Trust, Norway. The outpatient clinics are catchment area-based and cover a total population of approximately 200,000 people. Patient inclusion started in August 2014 and will continue until October 2016. Eligible for the study are outpatients who fulfill the *Diagnostic and Statistical Manual of Mental Disorders* (DSM-V) criteria for schizophrenia spectrum disorder (schizophrenia, schizoaffective disorder and schizophreniform disorder). Inclusion and exclusion criteria are presented in Table 1. Participation will be terminated if any of the exclusion criteria occurs (for instance pregnancy) during the trial or if the participant withdraws the informed consent.

**Table 1:** Inclusion and exclusion criteria

*Inclusion criteria*
Age between 18 and 67
Understand and speak a Scandinavian language
Fulfill the DSM-V criteria for schizophrenia spectrum disorder (schizophrenia, schizoaffective disorder and schizophreniform disorder)
*Exclusion criteria*
Pregnancy
Chest pain during exercise
Unstable angina pectoris
Recent myocardial infarction
Uncontrollable cardiac arrhythmia
Severe hypertension (>180/110 mmHg)
Comorbid diagnosis of mild mental retardation
Medical conditions incompatible with participation

*DSM Diagnostic and Statistical Manual of Mental Disorders*

#### Diagnosis

The diagnoses are based on the Structured Clinical Interview for Diagnostic and Statistical Manual of Mental Disorders (DSM-V) axis I disorders [40]. All interviewers engaged in diagnostics participate in supervised diagnostic evaluation meetings on a regular basis.

### Intervention

The exercise sessions will be conducted at the Buskerud and Vestfold University College and the computer gaming at a local high school in Tønsberg. Supervision of participants in the two groups is carried out by the same personnel employed in the outpatient clinics and dedicated to the project throughout the data collection period. The time spent with activities, as well as time spent with the intervention staff, is the same in both groups, and a specific time schedule is followed by the staff to ensure that each session in the exercise and comparison groups last 45 minutes. Participants in both groups are also accompanied by the project personnel during transportation, during the sessions of the intervention and during breaks.

The exercise sessions consist of supervised walking/running on a treadmill twice per week for 12 weeks. Each session will have the following structure; 8-minute warm-up, followed by 4x4 minute intervals with 85–95 % of maximum heart rate, with active pauses of 3 minutes walking/running at approximately 70 % of maximum heart rate. Heart rate will be measured and controlled continuously throughout the exercise session by research personnel with a heart rate monitor (RCX3 heart monitor from Polar (Polar Electro Oy, 90440 Kempele, Finland)) to ensure that the participants intervals and active pauses are performed with the desired intensity. The exercise session will end with a 5-minute cool-down period. The goal is to have the participants adhere to the protocol. However, we realize that some participants might have difficulties with this intensive program in the beginning of the intervention, and, thus, the protocol will be individualized and progressive both in terms of the number of intervals and the intensity. This high-intensity exercise performed as 4x4 minute intervals has previously been shown to be feasible, safe and effective for individuals with schizophrenia [41] and other clinical populations [21, 22, 26, 27, 38].

Participants in the comparison group take part in sessions performing computer-simulated sports activities (Nintendo Wii Sports (Nintendo, Frankfurt am Main, Germany) tennis, baseball, golf, bowling). Physiological responses (i.e. oxygen consumption, cardiac output, metabolic rate) of Nintendo Wii tennis (and baseball) has shown to be lower than brisk treadmill walking when tested in bouts of 10 minutes [42]. Through comparing neurocognitive and symptom scores in a group that is physically active with the performance of a group that simulates such activity, we expect to be able to extract the net effect of high-intensity exercise. Changes in simulated sports skills are monitored and continuous feedback is given to the participants by supervising personnel.

### Treatment as usual

All participants are registered outpatients at the psychiatric rehabilitation clinics in Tønsberg (NVDPS) or Larvik (SVDPS) in Vestfold County and receive individual treatment during project participation. The treatment is based on appointments at the clinic and/or home visits, and may include pharmacological treatment, individual psychotherapy, family interventions, and general psychosocial support through extensive collaboration with primary care health services. Episodes of illness exacerbation may require acute or planned admittance to local or county-level inpatient units, usually of short-term duration, while remaining registered at the outpatient clinic and continuing project participation.

### Feasibility study

A feasibility study was conducted from November 2013 through January 2014. Participants were outpatients at NVDPS Tønsberg and had present or previous psychotic symptoms. Apart from minor alterations, the same study protocol was followed as in the main trial. The intervention period was, however, considerably shorter, ranging from 3 to 6 weeks. Group assignment was not randomized in the feasibility study, and the raters were not blinded. Ten patients, 6 men and 4 women, aged 22–64 years (mean age 41) completed the study; 6 in the EG and 4 in the comparison group. The main purpose of the feasibility study was to gain experience with practical aspects of the protocol, evaluating its viability through particular focus on feedback from participants.

### Assessments

Participants will be assessed at three time points. The content of the assessments are outlined in Table 2. The first assessment occurs at baseline (T_0_) prior to randomization, because information from the baseline assessment is needed for validation of the inclusion and exclusion criteria and required for the stratification. The second assessment takes place immediately after completion of the intervention (T_1_). Longer-term outcomes or possible delayed effects not evident already at intervention cessation are tested in the third assessment 4 months post-treatment (T_2_).

**Table 2:** Assessments in the Effects of PHysical Activity in PSychosis (EPHAPS) trial

	Enrollment	Baseline	Randomization	Follow-up 1	Follow-up 2
TIME POINT		T_0_	0	T_1 12 weeks_	T_2 28 weeks_
ENROLLMENT					
Eligibility screen	X				
Informed consent	X				
Allocation			X		
INTERVENTIONS					
Exercise Group (EG)			>---------------<		
Computer Gaming Skills Group (CSG)			>---------------<		
ASSESSMENTS					
General assessments					
Sociodemographic data		X			
Changes in employment status				X	X
Information on tobacco, dietary habits and current level of physical activity		X			
Changes in use of tobacco, dietary habits, medication				X	X
Somatic health					
Physical examination: height; electrocardiography (ECG); medication record		X			
Weight, waist circumference, blood pressure		X		X	X
Changes in medication				X	X
Body composition assessment (bioelectrical impedance analysis/Tanita-weight)		X		X	X
Blood analyses: glucose, HbA1c, C-peptide, lipids (total cholesterol, HDL, LDL, TGA), homocystein, prolactin, T4, TSH, BDNF		X		X	X
Spirometry (pulmonary function test)		X		X	X
Cardiorespiratory fitness, physical activity and sleeping habits					
Maximal oxygen consumption		X		X	X
ActiGraph accelerometers (4 days)		X		X	X
Attitudes towards physical activity (PA)		X			
Change in attitudes towards PA (two selected items from Attitudes towards PA)				X	X
International Physical Activity Questionnaire (IPAQ)		X		X	X
Assessment of insomnia: Insomnia Severity Index (ISI)		X		X	X
Psychoactive substance use					
Selected parts of European Addiction Severity Index (EuropASI)		X		X	X
SCID section E		X			
Alcohol and Drug Use Disorder Identification Test (AUDIT and DUDIT)		X			
Alcohol and Drug Use Scale (AUS and DUS)		X		X	X
Fagerström test for Nicotine Dependence (FTND)		X		X	X
Saliva samples for assessing recent intake of tobacco, alcohol, illegal drugs and legal medication with abuse potential		X		X	X
Neurocognitive tests					
General Ability Index (GAI, Wechsler Adult Intelligence Scale-version 4)		X		X	X
The Emotional Biological Motion Test		X		X	X
Matrics Consensus Cognitive Battery (MCCB)		X		X	X
Diagnostics/symptoms/wellbeing					
The Structural Clinical Interview DSM-IV for axis I disorders (SCID-I)		X			
The Positive and Negative Syndrome Scale (PANSS)		X		X	X
The Psychotic Symptom Rating Scale (PSYRATS)		X		X	X
The revised Beliefs About Voices Questionnaire (BAVQ)		X			
Positive and Negative Affects Schedule (PANAS)		X		X	X
Depression: Calgary Depression Scale for Schizophrenia (CDSS)		X		X	X
WHO-5 Well-Being Index		X		X	X
Self-esteem (Ad Morrison)					
Clinical Global Impression Scale (CGI)		X		X	X
Psychosocial functioning					
Global Assessment of Functioning (GAFs and GAFf)		X		X	X
Quality of Life (Strauss Carpenter Level of Function (SCLOF))		X		X	X
Insight/adherence/apathy					
Insight of illness: Birchwood Insight Scale; cognitive insight: Beck Cognitive Insight Scale		X		X	X
Adherence to treatment: The Believes about Medicines Questionnaire (BMQ)		X		X	X
Apathy: Apathy Evaluation Scale (AES)		X		X	X

The table depicts specific time points in the trial for enrolment, intervention (groups and duration), and the assessments

*BDNF* brain-derived neurotropic factor, *HbA1c* glycosylated hemoglobin, *HDL* high-density lipoprotein, *LDL* low-density lipoprotein, *T4* thyroxine, *TGA* triglycerides, *TSH* thyroid stimulating hormone

Neurocognitive function is the primary outcome in the trial. Secondary outcome measures are positive and negative symptoms, wellbeing, as well as smoking habits, and somatic health parameters such as pulmonary function, body mass composition (e.g. body fat, bone mass), blood pressure, abdominal circumference, serum lipids, blood glucose. Brain-derived neurotrophic factor (BDNF), an important biomarker for neuroplasticity that seems to be upregulated after physical activity in schizophrenia [23] is also a secondary outcome measure. The changes in symptom level, wellbeing, neurocognitive scores and physiological (e.g. maximum oxygen uptake) and metabolic indices are assessed from T_0_ to T_1_ and from T_1_ to T_2_.

#### Symptoms, affect and wellbeing

Psychosis symptoms are assessed using the Positive and Negative Syndrome Scale for Schizophrenia [43]. Characteristics of delusions and hallucinations are specified using The Psychotic Symptom Rating Scale (PSYRATS, [44]), also targeting the distress dimension of these symptoms at baseline and changes therein between T_0_ and T_1_ and between T_1_ and T_2_ respectively. For endorsement of patients’ beliefs, emotions and behaviour related to auditory hallucinations, the revised Beliefs About Voices Questionnaire (BAVQ-R, [45, 46]) is applied. Participants’ depressive thinking is assessed by the Calgary Depression Scale for Schizophrenia (CDSS) [47, 48].

The degree of positive feelings towards oneself, life and future is not necessarily dependent on the occurrence and intensity of symptoms, even in the realm of severe, psychotic disorders. The dominating emotional tone of the present is assessed by The Positive and Negative Affect Schedule (PANAS), comprised of two statistically largely independent scales [49, 50]. The self-report is administered to all patients in both intervention groups at T_0_, T_1_ and T_2_, and, for measuring possible immediate effects, mid-intervention, after completing scheduled activity on a particular day, in addition to the PANAS assessment immediately after neurocognitive testing at T_2_. Self-esteem is assessed using a self-rating scale (Ad Morrison). General symptoms are appraised using the Clinical Global Impression Scale (CGI, [51]). Wellbeing is assessed using the 5-item self-report World Health Organization-5 (WHO-5) Well-Being Index [52]. The questionnaire has previously been used to monitor treatment in affective disorders [53].

#### Neurocognitive tests

Neurocognition is assessed with the Matrics Consensus Cognitive Battery (MCCB), which is developed particularly for assessment of treatment changes in schizophrenia. It assesses cognitive function in seven domains, including those most probable to increase as a function of increased cardiorespiratory fitness (executive functions, attention and memory) and offers parallel forms for repetitive testing. MCCB has been used in previous studies in Norway and the Norwegian Version has retained the original psychometric properties [54] with the possible exception of the test intended to measure social cognition. This subtest will be replaced by The Emotional Biological Motion Test [55], which is an experimental test newly developed by leading research groups in this field. At baseline participants will be examined with 6 subtests from the Wechsler Adult Intelligence Test-version 4, resulting in a General Ability Index-score equivalent to a full scale IQ.

#### Sociodemography

Basic sociodemographic data is collected, including sex, age, education, marital status, employment, living conditions, etc.

#### Lifestyle

Basic lifestyle parameters are assessed, including dietary habits, daily activity level, and daily use of stimulants containing caffeine and nicotine. Psychoactive substance use is thoroughly covered; see below.

#### Somatic health

A physical examination with medication record is undertaken in the trial. All participants are examined with electrocardiography (ECG). General health parameters such as weight, waist circumference and blood pressure, as well as pulmonary function and body composition are assessed. Blood samples, which encompass standard blood tests in addition to BDNF, will be collected from participants following overnight fasting at the three assessment points. For analysis of BDNF venous blood samples are collected in tubes containing ethylenediaminetetraacetic acid (EDTA). After centrifugation at 2500 g for 20 minutes, platelet-poor plasma will be isolated, aliquoted and stored at −80 °C. At the end of data collection, all samples will be analyzed for BDNF concentrations using enzyme immunoassay at the Research Institute for Internal Medicine, Rikshospitalet, Oslo, Norway.

#### Cardiorespiratory fitness, physical activity and sleeping habits

Maximal oxygen uptake (VO_2max_) is measured during a maximum exercise test on a treadmill using a modified Balke protocol [56]. Gas exchange is sampled continuously into a mixing chamber every 30 s by having the participants breathe into a Hans Rudolph 2-way breathing valve (2700 series, Hans Rudolph Inc., Kansas City, MO, USA) connected to a Jaeger Oxycon Pro gas analyzer (Erich Jaeger GmbH, Hoechberg, Germany), which measures the oxygen and carbon dioxide content.

Physical activity level is measured both objectively (ActiGraph accelerometer GT3X+ (ActiGraph, Pensacola, FL, USA) worn on the hip for 4 consecutive days; 2 weekdays and 2 days of the weekend) and subjectively using the International Physical Activity Questionnaire (IPAQ, short form [57, 58]). In addition, potential psychosocial mediators for change in physical activity and attitudes towards physical activity will be measured by previously developed and validated scales: social support for physical activity [59, 60], self-efficacy and outcome expectancies and attitudes [61, 62]. Sleeping habits are assessed using the ActiGraph data as well as the brief Insomnia Severity Index (ISI, [63]).

#### Psychoactive substance use

At baseline, a diagnostic assessment of psychoactive substance use is performed with the SCID section E (including alcohol, illegal drugs and legal drugs with abuse potential), and the severity of such use is additionally measured with the Alcohol and Drug Use Disorder Identification Tests (AUDIT [64] and DUDIT [65]). Assessments at 3 time points (T_0_/T_1_/T_2_) will include selected parts of the European Addiction Severity Index (EuropASI) [66], Alcohol [67] and Drug Use Scales [68] (AUS and DUS respectively), Fagerström test for Nicotine Dependence [69] and saliva samples for assessing recent intake of tobacco, alcohol, illegal drugs and legal medication with abuse potential. The toxicological analyses will be performed at Division of Forensic Toxicology and Drug Abuse Research, Norwegian Institute of Public Health.

#### Psychosocial functioning, adherence and insight

Strauss Carpenter Level of Functioning Scale (SCLOF) is utilized for the endorsement of patients’ social and work functioning. Global Assessment of Functioning (GAF, [70]) is divided into two scales measuring symptoms (GAFs) and function (GAFf) to improve psychometric properties [71]. Insight of illness (Birchwood Insight Scale, IS, [72]), cognitive insight (Beck Cognitive Insight Scale, BCIS, [73]) and apathy (Apathy Evaluation Scale, AES, [74]) are assessed using self-rating scales.

### Protocol deviations

When absent from scheduled exercise or computer gaming, the participant will be contacted and offered to participate on a following day the same week. Participants who fail to complete at least 60 % of the total number of intervention sessions or have not participated for more than 2 successive weeks are considered protocol violators. Participants who are unable to meet for the VO_2max_ test within 10 days following the last training session will also be considered protocol violators. However, they will be allowed to stay in the study and will be tested as soon as possible, as well as invited to take part in T_2_. Statistical analyses will be performed both based on the principle of “intention-to-treat” including everyone regardless of protocol violations, but “per protocol” analyses will also be executed, comparing only those who completed the two treatment conditions according to the protocol.

### Sample size calculations

We assume that the outcome measures are continuous variables with a normal distribution, and two independent clinical groups are being compared. Estimates of statistical power are calculated for the primary outcome measure. The neurocognitive tests applied in the study (MCCB battery) are designed for repeated administrations to test possible treatment effects. Some improvement must be expected in both groups because of the treatment/stimulation and as retest effects (being accustomed to the test design if not the actual test material). Nevertheless, we expect that the effect size in the EG will be 0.50 standard deviations (which is a medium effect size) *larger* than the increase in the CSG, which given a statistical power of 0.80 and alpha of 0.05 necessitates a sample size of 55 in each group. The dropout rate in the current study after randomization and group assignment is estimated to 15 %. Thus, the estimate is that recruitment of 126 participants is needed to secure the participation of 55 individuals in each group. Further attrition at later stages in the study progress is accounted for by utilizing the “intention-to treat” analysis.

### Randomization and stratification

After the baseline assessments, the participants will be randomly assigned to either CSG or EG. A computerized random number generator is utilized to produce the allocation sequence. Equal distribution of participants in the two groups on CRF is ensured by stratification on expected median with regard to scores on VO_2max_ at baseline. By varying the size of the stratification blocks, each treatment assignment remains unpredictable. Senior researchers in the project (JAE, JE) generated the randomization sequence. A dedicated project coordinator, who is not located near the participants during sessions and not concerned with outcome assessments, administers the group assignment using the method of concealed envelopes. The name, date of birth, trial number and assigned treatment is registered on a log sheet made for the participant in the trial.

### Statistical analysis

Decisions about sample size and design of the project are primarily aimed at comparing the EG with the CSG. The main statistical analyses will be variants of analyses of variance such as Multivariate ANOVA (MANOVA) to test overall group differences, repeated measures ANOVA to look for interactions between time and group (i.e. to test whether the EG have larger change between time points than CSG). Changes in outcome measures will be correlated with changes in CRF. Analysis of covariance will be used to test hypotheses whether the effect of specific variables extend beyond effects of nonspecific variables. Baseline data could be analyzed without referral to later group membership, enabling analyses of empirically derived subgroups such as participants scoring low or high on the physiological measures, or participants with or without concurrent tobacco use. Baseline data will also be analyzed with regression analyses and correlations/partial correlations controlling for potential confounding variables.

### Data storage and use of statistical tools

The data will be stored and analyzed in the Vestfold Hospital Trust using the Statistical Package for the Social Sciences (SPSS version 18.0.1, SPSS inc., Chicago, IL, USA). Data cleaning will be performed via SPSS syntax operations (SPSS Inc., Chicago, IL, USA).

### Ethical considerations

The project is approved by the Regional Ethics Committee of Southern and Eastern Norway (REK Sør-Øst) under file number 2014/372/REK SØR-ØST C. Trial registration has been carried out at ClinicalTrial.gov (NCT02205684). Informed and written consent is a pre-requisite for participation. Initial information about the study will be given to eligible patients by the regular staff during a routine appointment in the outpatient clinic, or by a project co-worker. Further detailed written and oral information about the trial will then be given by the project co-worker. It will be assured that participation is voluntary, that the participants can withdraw at any time point and will receive ordinary treatment whether they choose to participate or not. If the patient understands the nature of the research and is willing to participate, he or she will be asked to sign a consent form. The interviews in the trial will be completed in several encounters with the participant, and emphasis will be put on avoiding unnecessary strain on the individual and adjustment to own preferences. Similarly, when taking part in the treatment intervention, attention will be paid to the physical fitness of the participant. Potential alteration of the intensity and frequency of the physical activity will be adjusted individually over several weeks. Participants experiencing discomfort or possible medical complications while exercising on the treadmill will receive immediate necessary examination by medical staff in the unit. Positive as well as negative results will be published according to the Consolidated Standards of Reporting Trials (CONSORT) guidelines [75].

### Motivational work

Motivating patients with severe mental disorders, prone to be insufficiently physically fit to perform high-intensity interval training twice a week, is perceived a major challenge. Although the motivational work is performed by the intervention staff in informal settings when talking to participants before and after each session, as well as during transportation between their home and the project facilities, it needs to be structured and theory-based. Key constructs in social cognitive theory [76] are applied (i.e. opportunities to perform physical activity; social support for physical activity; confidence to do physical activity; expected benefits and costs of performing physical activity; knowledge and skill to perform physical activity; personal goal setting and monitoring of physical activity), putting much effort in translating this knowledge into effective practices.

### Adverse events

Medical examination and ECG are performed before the exercise program starts, and all patients are evaluated as suitable for training. One potential concern might be the combination of physical exercise and medication, but there is no evidence for danger in exercising while using therapeutic doses of psychotropic medication [77].

### Emergency procedures

To minimize the risk of medical emergencies, patients with a known unstable heart condition (e.g. unstable angina pectoris and recent myocardial infarction) will be excluded from the study. Also, ECG and physical examination performed by a physician are performed before inclusion in the study. Health staff with specific knowledge of cardiopulmonary resuscitation will be present at all treadmill-sessions of physical testing and exercise. In case of symptoms such as chest pain and dizziness during exercise, the session will be discontinued immediately. In case of symptoms of cardiac arrest (unresponsive person with no or abnormal breathing), cardiopulmonary resuscitation will be initiated immediately and an ambulance will be called.

## Discussion

Physical activity has been shown to reduce symptoms of depression and increase cognitive functions in other groups associated with cognitive dysfunction such as Parkinson’s disease [78, 79] and old age [80–82]. It is, therefore, plausible that physical activity may have beneficial effects, both on the level of mental symptoms and cognitive function in schizophrenia, but high-quality data from RCTs is lacking. In the present study high-intensity treadmill exercise for 12 weeks is compared to computer-based skills training, otherwise shown to have beneficial effects on cognitive function [33]. Well-validated measures of the respective functional domains serve as main outcome measures and assessments are performed pre-training, immediately after completed training and 4 months post-treatment allowing appraisal of possible delay between increased CRF and reduced symptoms. Secondary outcome measures are related to factors reducing risk of metabolic syndrome and improving physical health. If we find that intensive exercise can reduce symptoms and increase cognitive performance, it may have implications for future treatment of patients with schizophrenia. As cognitive remediation programs so far have focused on learning techniques in a top-down fashion [83], documenting possible bottom-up beneficial effects may open up new routes for helping these patients increase their cognitive functions. In regard to symptom reduction, broadening the armamentarium of methods, primarily consisting of pharmacological treatment and psychotherapy, to also contain physical activity, will be welcomed as increased CRF in general has an effect on both morbidity and mortality.

As the general optimism regarding the positive effects of physical activity on symptoms and cognition has now spread from normal aging, treatment of depression to schizophrenia, resulting in small-scale or non-systematically researched interventions, the findings of no differential increase in mental and cognitive functions, may lead to some disappointment regarding the role of physical activity in treating these elements of severe mental disorder. Early death from cardiovascular disorders and reduced function due to low cardiovascular capacity and low muscular strength are other important treatable elements of the schizophrenia group of disorders. It is important that treatment approaches are empirically-based and not merely plausible inferences from knowledge in other groups. High-intensity exercise is demanding for the patient, as well as the help-giving system. Thus, with this level of knowledge, emphasis should be placed on finding causal effects or mediating mechanisms, while the cost-effectiveness of physical activity, as a way to improve functions other than key symptoms in schizophrenia, is another important issue.

Perhaps, the greatest challenge conducting an intervention study of this kind is making the participants adhere to the demanding exercise regime. To meet this challenge, participants are given individual tutoring and motivational talks and are offered project-initiated transportation in order to increase adherence – a support regime which is equally comprehensive in both groups. Thus, the present trial involves a support system that would not be sustainable in ordinary clinical work.

In selection of an adequate control condition, it is important for adherence to the skills training control group that both the participants and the intervention staff find the activity meaningful, and by that preventing an expectancy-related lack of effect in the comparison group.

## Trial status

The recruitment started August 2014. By April 2015 a total of 32 subjects were participating. Five subjects have dropped out of the study. The planned recruitment of 126 participants will be completed by October 2016.

## Abbreviations

AES: Apathy Evaluation Scale
ANOVA: analysis of variance
AUDIT: Alcohol Use Disorder Identification Test
AUS: Alcohol Use Scale
BAVQ-R: The revised Beliefs About Voices Questionnaire
BCIS: Beck Cognitive Insight Scale
BDNF: brain-derived neurotrophic factor
BMQ: The Believes about Medicines Questionnaire
CDSS: Calgary Depression Scale for Schizophrenia
CSG: Computer Gaming skills Group
CGI: Clinical Global Impression Scale
CONSORT: Consolidated Standards of Reporting Trials
CRF: cardiorespiratory fitness
CVD: cardiovascular disease
DSM-V: *Diagnostic and Statistical Manual of Mental Disorders*
DUDIT: Drug Use Disorder Identification Test
DUS: Drug Use Scale
ECG: electrocardiogram
EDTA: ethylenediaminetetraacetic
EG: exercise group
EPHAPS: Effects of Physical Activity in PSychosis
EuropASI: European Addiction Severity Index
FTND: Fagerström Test for Nicotine Dependence
GAF: Global Assessment of Functioning
GAI: General Ability Index
HIIT: high-intensity interval training
IPAQ: International Physical Activity Questionnaire
IS: Insight Scale
ISI: Insomnia Severity Index
MANOVA: multivariate analysis of variance
MCCB: Matrics Consensus Cognitive Battery
NTNU: Norwegian University of Science and Technology
NVDPS: Nordre Vestfold Distrikt Psykiatrisk Senter
PANAS: Positive and Negative Affects Schedule
PANSS: Positive and Negative Syndrome Scale
PSYRATS: The Psychotic Symptom Rating Scale
RCT: randomized controlled trial
REK Sør-Øst: Regional Ethics Committee of Southern and Eastern Norway
SCID-I: The Structural Clinical Interview DSM-IV for axis I disorders
SCLOF: Strauss Carpenter Level of Functioning Scale
SVDPS: Søndre Vestfold Distrikt Psykiatrisk Senter
VO_2max_: maximal oxygen uptake
WAIS: Wechsler Adult Intelligence Scale
WHO: World Health Organization
